# Lights and Shadows in Hepatic Encephalopathy Diagnosis

**DOI:** 10.3390/jcm10020341

**Published:** 2021-01-18

**Authors:** Piero Amodio, Sara Montagnese

**Affiliations:** Department of Internal Medicine, DIMED, University of Padova, I-35100 Padova, Italy; sara.montagnese@unipd.it

**Keywords:** liver failure, encephalopathy, delirium, coma, cirrhosis

## Abstract

Hepatic encephalopathy (HE) is a form of brain dysfunction that is caused by liver insufficiency and/or portal-systemic shunting. The exact nature of HE is debated; as such, conflicting uses of the term “HE” may cause inconsistencies in its detection and management. This review highlights the meaning of the term “HE” on the basis of its historical origins and current consensus. It also provides criteria for the diagnosis of the condition based on its phenotypes and risk factors for its occurrence. The procedure for differential diagnosis from other conditions which result in similar phenotypes is considered, together with precipitants and confounders. Finally, the current multidimensional approach for the correct clinical reporting of HE episodes is discussed.

## 1. Introduction

The diagnosis of hepatic encephalopathy (HE) is relevant as it is a marker of poor survival in cirrhosis and acute liver failure (ALF) [1,2], and is a disabling condition resulting in poor quality of life for patients and their caregivers [3]. Further, HE incurs significant direct costs to health service systems [4], as it is the second leading cause of hospitalization in patients with cirrhosis and the primary reason for re-hospitalization [5]. It also results in considerable indirect costs related to loss of work for patients and caregivers [6]. A correct diagnosis is required in order to select proper treatment, prevent further episodes in individual patients, and conduct meaningful prevention/treatment trials.

While the diagnosis of HE may seem simple (and frequently this is the case), the diagnosis can be complex and uncertain. The issue is not trivial and firstly depends on what one considers to be HE; i.e., on its definition.

## 2. The Meaning of the Term “HE”

The recognition of an association between jaundice and behavioral alterations is ancient, while that between cirrhosis and confusion/stupor is some three centuries-old, and the pathophysiological explanation for these associations dates back to end of the nineteenth century [7].

On this basis, the American Association for the Study of Liver Disease (AASLD)/European Association for the Study of the Liver (EASL) practice guidelines for HE define HE as “*Brain dysfunction caused by liver failure and/or portal-systemic shunting (PSS); it manifests as a wide spectrum of neurological or psychiatric abnormalities ranging from subclinical alterations to coma*” [8]. The term “hepatic” is used to underline a specific pathophysiological link to liver failure and/or portal-systemic shunting.

However, the habit of calling encephalopathy “*hepatic*” on the basis of a “clinical suspicion” in patients with liver disease remains [9,10,11,12]. This is open to criticism, since seldom in medicine is encephalopathy qualified based on the disease in which it occurs. By contrast, it is generally qualified based on the pathophysiological mechanism causing encephalopathy/delirium in order to direct etiological treatment. Thus, the terms “*lung encephalopathy*” or “*cardiac encephalopathy*” do not exist, while the terms “*hypercapnic encephalopathy*”, “*hypoxemic encephalopathy*”, and “*cerebral hypoperfusion encephalopathy*” are used.

Thus, if “*hepatic*” does not refer to a specific mechanism, it would be better replaced by a term that does (i.e., “patient with cirrhosis and benzodiazepine intoxication”, “opioid overdose”, “hyponatremic encephalopathy”, “septic encephalopathy”, or “hyperammonemic encephalopathy”, etc.). This would avoid misinterpretation and mismanagement due to the use of the same treatment for conditions with different underlying types of pathophysiology.

As an analogy, using the term HE in a broad meaning would be the same as calling fever in patients with cirrhosis “*hepatic fever*”, regardless of its cause (i.e., pneumonia, urinary tract infection, spontaneous bacterial peritonitis, etc.), and treating it in the same way. The idea that in patients with cirrhosis all encephalopathies should be classified and managed depending on their etiology was clearly formulated by Riddell around 65 years ago [13]: “*… among a group of patients with severe liver disease a number of neurological disturbances will be met with; not all of these are the disease known as hepatic coma. Among these other states are the psychoses associated with chronic alcoholism and nicotinic acid deficiency, electrolyte disturbances, septicaemia, increased response to narcotics and subdural haematoma*.”

Considering the definition given by the AASLD/EASL practice guidelines, the following question may arise: “*Which type of brain dysfunction is caused by liver failure and/or PSS?*”. This question implies the detection of a mechanism that *specifically* links these conditions. Such a link between a failing liver and/or PSS and encephalopathy concerns abnormalities in nitrogen metabolism, as the liver has a unique role in the detoxification of ammonia and most other substances coming from the gut. This has been proven by the observation of dogs undergoing portal-caval shunting who (1) developed encephalopathy after the consumption of ammonia salts and nitrogen-containing-foods, (2) reduced their urinary urea excretion, and (3) reduced their capacity to synthetize urea from gastric-infused carbamic acid [14]. Further, the oral administration of ammonium chloride to cirrhotic patients causes coma [15], and the toxicity of ammonia to the human brain has been proven by cognitive defects with respect to attention/executive function and coma in individuals with hereditary defects in urea cycle enzymes [16]. Finally, the creation of large portal-systemic shunts causes hyperammonemia and encephalopathy in humans, and shunt obliteration reduces ammonemia and improves HE [17].

PSS and hepatic failure may cause an increase in any neurotoxic substance originating from the gut that has a high first-pass hepatic metabolism, like ammonia. Research on this has been limited over the past years, after emphasis was given to the topic by Zieve [18]. Substances with the above features which may have a pathophysiological role in HE include: (1) manganese (particularly in the motor disturbances associated with HE), since this heavy metal deposits in the basal ganglia due to its reduced clearance in portal-systemic shunting and cholestasis [19], and (2) indole, that crosses the blood–brain barrier and produces oxindole within the brain, which is a neurotoxic substance [20]. Further, gut dysbiosis, Kuppfer cell dysfunction [21], and portal-systemic shunt may favor systemic inflammation, which also affects brain function [22,23].

It should be emphasized, however, that despite hyperammonemia being a necessary condition for the occurrence of HE (and thus subjects without hyperammonemia should be suspected of having a delirium of alternative origin), hyperammonemia is common in cirrhosis, mainly because of PSS [24], and its occurrence does not imply a phenotypical HE expression. Indeed, ammonia interacts with other factors to disturb brain function [25]. This explains why the clinical expression of HE is only roughly related to ammonia plasma levels, since individual susceptibility to developing a hyperammonemia-related phenotype depends on several factors. Thus, in the same individual, changes in co-factors may change the effect of ammonia, and varying brain susceptibility to the levels of ammonia was proven long ago [15]. More recently, an elegant study by Shawcross and co-authors [26] proved the different effects of ammonia depending on cytokine levels. We ourselves have shown that same levels of ammonia have different effects on brain electrogenesis depending on sodium levels [27].

This limits the value of using absolute isolated ammonia levels as an index of HE. However, the diagnostic value of plasma ammonia is high, since normal ammonia levels suggest that the degree of liver failure/shunt is insufficient to support a working diagnostic hypothesis of HE. A recent study [11] seems to contradict this view, showing that recovery of normal mental state is the same in patients with delirium and ammonia levels higher or lower than 75 mmol/L (the upper limit of normality) [24]. Of note, 80% of these patients had a history of overt HE. However, the patients with low ammonia levels had a higher rate of infection than the others. In addition, in all patients the precipitating factor of the HE episode was treated, and it is reasonable to expect that this would have resulted in clinical improvement. The use of lactulose in all patients was probably irrelevant to the conclusions of the study, since it is useful in patients with HE and innocuous in those with other kinds of delirium.

HE in acute liver failure is a distinct type of HE [8] that occurs in the context of systemic hemodynamic alteration and multi-organ failure (MOF) [28,29]; thus, it has separate features in which brain swelling due to acute hyperammonemia and intracranial hypertension have a peculiar role.

Recently, it has been observed that HE in acute-on-chronic liver failure also has some peculiar features [30,31]. It frequently occurs in sepsis, cytokine storm, and MOF that reasonably produce overlapping metabolic/hemodynamic encephalopathies. These may deserve to be considered separately and frequently require multitarget treatment in managed intensive care unit (ICU) patients.

In all cases of encephalopathy, especially in the context of ALF or acute-on-chronic liver failure (ACLF), the exclusion of alternative causes is mandatory because an incorrect, missed, or delayed alternative diagnosis (e.g., brain hemorrhage) has profound consequences.

## 3. The Diagnosis of HE

The diagnosis of HE, as with every clinical diagnosis, results from the a priori probability of HE before any observation, and the probability that a clinical finding relates to HE. This should be compared with the probability of alternative conditions.

Thus, the degree of certainty for the diagnosis of HE depends on three key steps: (1) the a priori probability of HE, (2) the recognition of a clinical pattern suggestive of HE, and (3) the consideration of alternative conditions.

In formal Bayesian terms:

*Odds of HE = a priori odds of HE prevalence × positive likelihood ratio of clinical findings for HE vs. a priori odds of alternative condition prevalence × positive likelihood ratio of clinical findings for alternative conditions.*

However, formal Bayesian estimations cannot be performed, because an exact quantification of the parameters of the equation is not available and varies reasonably depending on the clinical settings. However, the clinician’s reasoning is still Bayesian and the related questions are as follows: Is the presentation suggestive of HE? Is it probable that in this patient HE may occur? Are there other conditions that may explain this clinical presentation?

The a priori probability of HE depends on the severity of liver failure [32,33] and/or the extent of PSS [34], in addition to the history of previous episodes of overt HE [27]. Recently, a clinical score was proposed to assess the risk for the first episode of overt HE [11].

Of note, the severity of liver failure and PSS have a fundamental role, and they are associated with high levels of plasma ammonia. This is a known risk factor for HE [35,36].

The a priori probability is increased by the occurrence of precipitating factors for HE, even if their prevalence varies considerably between the studies (Table 1).

*Precipitating factors* can be considered as those conditions which intervene in the pathophysiology of HE by increasing the production of ammonia, reducing its disposal or increasing its neurotoxicity. For instance, gastrointestinal bleeding and constipation increase ammonia production, and inflammation (especially that associated with infections) and hyponatremia increase ammonia toxicity [26,27,40]. Of note, infections, as well as hypothyroidism, increase ammonia production [13,37].

**Figure 1:**
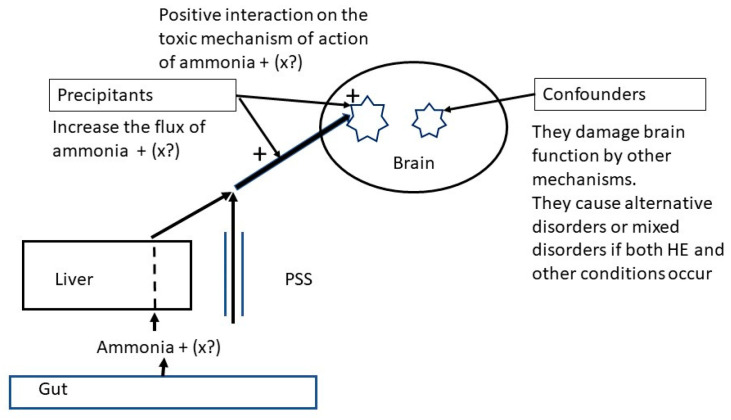
Schema showing the different concepts of precipitants and confounders.

In contrast to precipitants, conditions that alter brain function by a direct mechanism, independent of liver nitrogen metabolism, can be called *confounders* (Figure 1).

It should be considered that the distinction between precipitants and confounders is not always simple or clear. Some factors such as sepsis, hyponatremia, and hypothyroidism, which function as precipitants (because they intervene in the mechanisms of ammonia production or toxicity), can also alter brain function per se, as occurs in patients without liver failure [41,42,43,44]. Treatment should be directed at these factors and not only at hyperammonemia [8].

The recognition of the clinical presentation that HE is an obvious pre-requisite for its diagnosis. HE may manifest as coma, delirium of various degrees (mainly sedated, but sometimes agitated) [8], and sometimes with transient focal symptoms [45]. Further, HE may present as an almost continuous cognitive dysfunction, interspersed with more or less severe episodes of delirium [8]. A mild presentation of HE mimics persistent or episodic non-amnestic minimal cognitive dysfunction or disinhibition [46]. Finally, motor signs can be associated or (rarely) dominate the presentation. The most obvious and common is negative myoclonus, which produces the classical finding of asterixis. Other motor findings are extrapyramidal manifestations such as parkinsonism [47], chorea and hemiballismus [48], or spastic paraparesis [49]. These symptoms, generally found in association with large PSS, are called non-Wilsonian hepatocerebral degeneration and hepatic myelopathy [46]. These conditions, however, can be considered subtypes of HE as both are superimposed, and portal-systemic shunting which intervenes in their pathophysiology and overlap with mild confusion is the rule (Table 2).

Accordingly, for the differential diagnosis of the clinical findings compatible with HE [50], one should briefly consider the probability of alternative/overlapping causes of the clinical findings: coma, delirium, transient focal attacks, persistent undulating dementia-like mental decay, and motor disorders [51,52,53,54,55]. The occurrence of sudden coma requires brain imaging, since hemorrhagic stroke has an increased prevalence in liver failure [56]. Similarly, the occurrence of persistent/highly recurrent HE may require the search for additional/alternative causes of dementia or neurodegenerative disorders by brain imaging and extensive investigation.

Of note, the occurrence of a finding compatible with HE and a high a priori probability does not exclude the existence of mixed encephalopathy. Indeed, the addition of various factors damaging brain function facilitates the occurrence of symptoms. In the case of a mixed encephalopathy, the treatment of the brain dysfunction caused by HE may result in an improvement, but does not completely revert the clinical picture [57].

**Figure 2:**
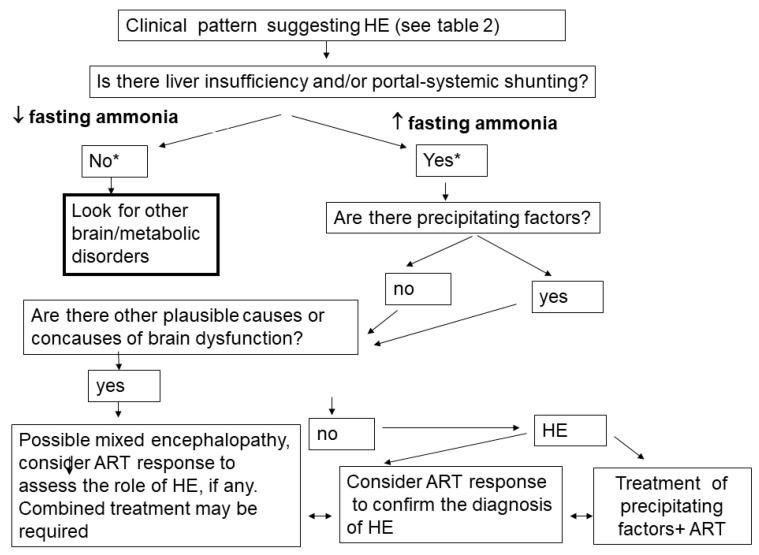
Flow chart for the diagnosis of hepatic encephalopathy (HE) (modified from [50]). ART: ammonia-reducing treatment (non-absorbable antibiotic ± disaccharides). * Low ammonia is considered to have high negative predictive value for HE [8], since it suggests: (1) relatively good liver function, (2) negligible portal-systemic shunting, and (3) negligible gut dysbiosis in cirrhosis.

A chart that summarizes the diagnostic process is reported in Figure 2.

Additionally, if HE is present and is the only cause of the clinical findings (thus, *what is HE and what is not HE*), the diagnosis of HE requires some additional attributes relating to its type, severity, rate of recurrence, precipitant, and facilitating factors [58]. Thus, a complete diagnosis should be multidimensional (as was first suggested by Ferenci et al. [10] and emphasized in the practice guidelines of the AASLD/EASL [8] and the Italian association for the Study of the Liver AISF [58]).

Of note, recent brain imaging techniques [59,60,61] and accurate microbiome and metabolomic studies [62] are providing important new insights on HE, but at the moment these techniques are limited to research purposes.

Conflicting opinions concern the diagnosis and quantification of mild forms of HE. In the 1950s Parsons-Smith et al. [63] recognized two important findings: (1) some patients with cirrhosis may have subtle mental changes and psychometrical alterations in the absence on unequivocal neurological signs, and (2) patients without any alterations may have EEG abnormalities. These authors clearly showed that HE can produce (1) mild equivocal mental/cognitive/behavioral alterations, and (2) even neurophysiological alterations without any other evidence. Conn et al. [64] provided a quantification of mental changes in HE, slightly modifying that of Parsons-Smith et al. [63], and classified grade 1 HE as a condition characterized by subtle cognitive/behavioral alterations without disorientation (in time or space). Since then, new techniques of investigation of cognitive investigation (based on techniques requiring patient cooperation) and brain function (i.e., independent of patient’s cooperation) have been developed, with a plethora of definitions and names provided, including latent HE, subclinical HE, minimal HE, and grade 1 HE. Basically, there is an agreement that (1) some patients do not show any clinical symptoms/signs of HE but do have neurophysiological or psychometrical alterations, and (2) other patients have “something wrong” in their awareness, behavior, or attention that can be recognized by skilled clinicians and caregivers. The former condition is preferably called minimal HE, whereas the latter is grade 1 HE. However, since both conditions are not unequivocal (at odds with frank disorientation) and the line between the two depends on the sensitivity of the observer, a proposal was made [8,65] to gather the two conditions under the heading of “covert” HE (mainly because the sound “covert” evokes the antonym of “overt”), considering that this condition requires quantification by objective tests. This proposal aimed to avoid possible inter-observer disagreement and inconsistency in the detection of grade 1 HE. At any rate, possible confusion arises if “covert” is misinterpreted as a synonym of minimal, or if mildly symptomatic HE (grade “1”) is considered to overlap with asymptomatic HE (i.e., minimal HE). Thus, conflicting opinions still exist about the use of the terms.

Operative definition for the diagnosis of grade 1 HE was provided by Vilstrup et al. [8]. The diagnosis of minimal HE requires proper testing. This can be neurophysiological, (e.g., quantified electroencephalogram, evoked potentials, etc.), psychophysiological (e.g., critical flicker frequency), or neuropsychological [66,67]. Of note, psychometrical testing needs to be oriented to the cognitive domains mostly altered by the initial phase of HE (e.g., cognitive speed, divided attention, sustained attention, inhibition, working memory) and be properly standardized for single patients, since cognitive performance depends on age and education and, most probably, geographical/national factors [66]. Thus, locally standardized Z-score based techniques, such as the psychometric HE score PHES [68], are preferable. Recently, a very rapid oral technique based on animal naming has been suggested [69], but needs confirmatory studies. Computerized tests based on chronometric techniques have been used [70,71,72] and may be more repeatable since they are based on the repetition of many trials, but only well-educated, cooperative individuals can be easily tested. Further, any kind of mild psychometrical/neurophysiological dysfunction in cirrhosis cannot be immediately attributed to minimal HE, since mild cognitive dysfunction can have many concurrent causes [73,74]. 

## 4. Conclusions

The existence of liver failure, PSS, prior HE, frailty, and precipitating factors increase the risk of HE. However, the risk provided by these conditions (with varying degrees) cannot be exactly quantified, and neither can the risk provided by their interaction. At present, therefore, they may only confer a subjective degree of confidence relating to the a priori likelihood for the diagnosis of HE.

Another milestone for diagnosis depends on the recognition of the phenotype of HE [50], which is unspecific. Thus, alternative or concurrent conditions should be considered, particularly for patterns where a rapid alternative diagnosis may have high relevance with regard to the outcome (for example coma). Other conditions that require intensive work are the presentations characterized by prominent motor features or persistent fluctuating mental alterations. In most cases the diagnosis is simple, particularly when there is strong a priori probability that the patient only has liver disease and the phenotype of HE is one of delirium. In other conditions, the diagnosis can be more challenging. It is reasonable to assume that HE can be confirmed if a full-dose regime of non-absorbable disaccharides and non-absorbable oral antibiotics (i.e., a regimen significantly reducing plasma ammonia) improves or completely reverts symptoms in a few days. Finally, after having reached a correct diagnosis of the existence of HE, multidimensional qualification is required to characterize the type, severity, time course, and precipitant-favoring factors, in accordance with present practice guidelines.

## Figures and Tables

**Table 1:** Prevalence (percentage and 95% CI) of precipitating factors for hepatic encephalopathy (HE).

	Ali et al. [37] *N* = 100	Cordoba et al. [31] (*N* = 460)	Devrajani et al. [38] (*N* = 87)	Stauss et al. [39] (*N* = 168)
Infections	20 (13–29)	26 (22–30)	67 (56–76)	31 (24–38)
Bleeding	14 (8–22)	11 (9–14)	45 (35–55)	20 (15–27)
Constipation	37 (28–47)	-	40 (39–60)	
DDEI *	70 (60–78)	89 (86–91)	54 (44–64)	36 (29–44)
Acute alcoholism		17 (14–20)	-	2 (1–6)
Not recognized	11 (6–19)	-	6 (2–13)	10 (6–15)

DDEI *: Dehydration, diuretics, electrolyte imbalance; CI: confidence interval. Note: the sum of the percentages may be higher than 100 because of overlap.

**Table 2:** Phenotypes of HE.

Pattern		Description
(A) Coma (grade 4 West Haven classification)		The patient’s eyes are closed; they are unresponsive even to painful stimulation.
(B) Rapidly developing state of confusion (delirium)(grade 2–3 according to the West Haven classification)	Inhibited	The patient is more or less disoriented in time and/or space and/or identity and is more or less somnolent/stuporous. Asterixis is usually detectable.
	Agitated	The patient is disoriented in time and/or space and/or identity and is agitated/angry/restless. Asterixis is usually detectable
(C) Almost continuous mild mental dysfunction with interspersed recurrent episodes of confusion(persistent/almost continuous HE with frequent relapses)		The pattern is dementia-like. Asterixis is usually detectable.
(D) Predominant motor disorder with mild/moderate mental dysfunction/confusion(corresponds to the conditions of hepatic parkinsonism and hepatic myelopathy/non-Wilsonian hepatocerebral degeneration)	Extrapyramidal	Parkinsonism, chorea, or athetosis. Asterixis is usually detectable.
	Pyramidal	Spastic paraparesis with hyperreflexia. Asterixis is usually detectable.
(E) Mild brain dysfunction		The patient is oriented for time and space and his/her mental activity seems normal/near-normal; however, caregivers or heath personnel may recognize a deterioration of the patient in terms of behavior, irritability, and cognition.Upon psychometric testing, alterations are detectable (related to attention, working memory, cognitive speed, and inhibition). Other signs, associated or independent of psychometrical alterations, include slowed electroencephalographyc activity and/or reduced critical flicker frequency. Dissociations between the techniques are frequent.

## Data Availability

No new data were created or analyzed in this study. Data sharing is not applicable to this article.

## Abbreviations

HE	Hepatic encephalopathy
ALF	Acute liver failure
AASLD	American Association for the Study of Liver Disease
EASL	European Association for the Study of the Liver
PSS	Portal-systemic shunt
ACLF	Acute-on-chronic liver failure
AISF	Italian Association for the Study of the Liver
ICU	Intensive care unit
DDEI	Dehydration/diuretics/electrolyte imbalance
CI	Confidence interval
MOF	Multi-organ failure
